# Primary Aldosteronism Subtype Diagnosis: Adrenal Venous Sampling vs. CXCR4 PET CT

**DOI:** 10.1002/edm2.70254

**Published:** 2026-09-07

**Authors:** Shengzhuo Liu, Yifan Zhang, Xiaoyang Liu, Huijun Zhou, Sikui Shen, Ning Yin, Yuchun Zhu

**Affiliations:** ^1^ Urology Department, West China Hospital Sichuan University Chengdu China; ^2^ West China School of Medicine Sichuan University Chengdu Sichuan China; ^3^ Department of Nuclear Medicine, West China Hospital Sichuan University Chengdu Sichuan China; ^4^ Zizhong County People's Hospital Neijiang City Sichuan China

**Keywords:** adrenal venous sampling, CXCR4‐directed ^68^Ga‐pentixafor PET/CT, primary aldosteronism, subtype differentiation

## Abstract

**Purpose:**

To explore the clinical value of adrenal venous sampling (AVS) and CXCR4‐directed ^68^Ga‐pentixafor PET/CT in the subtype diagnosis of primary aldosteronism (PA), with postsurgical blood pressure control and biochemical cure as primary endpoints, and to compare the accuracy and practicability of the two methods.

**Methods:**

A retrospective cohort study was conducted in PA patients undergoing unilateral adrenalectomy at West China Hospital between February 2023 and June 2025. Patients were divided into the AVS and CXCR4 imaging groups. The primary outcome was the clinical and biochemical success rate according to the Primary Aldosteronism Surgical Outcome (PASO) criteria. In the subgroup with both procedures, AVS was considered the reference standard to assess the diagnostic efficacy of CXCR4.

**Results:**

A total of 160 patients completed the trial. According to the PASO criteria, the total biochemical success rate was 93.1%, among which the complete biochemical success rate in the AVS group was 84.1%, and in the CXCR4 group it was 93.1% (*p* = 0.091); the total clinical success rate was 82.5%, with a complete clinical success rate of 40.9% in the AVS group and 30.3% in the CXCR4 group (*p* = 0.412). There were no statistically significant differences between the two groups. In 16 patients with both examinations, CXCR4 showed a sensitivity of 70.0%, specificity of 83.3%, accuracy of 75.0%, and moderate agreement with AVS.

**Conclusion:**

CXCR4 showed no statistical difference from AVS in diagnostic and prognostic outcomes in this study. However, discordance in multinodular disease indicates that molecular imaging cannot fully reflect the heterogeneity of aldosterone secretion. This finding requires verification in a larger cohort.

## Graphical abstract

We compared CXCR4 PET/CT and AVS for primary aldosteronism subtyping. CXCR4 showed no statistical difference from AVS in diagnostic and prognostic outcomes in this study, which offers a non‐invasive alternative for PAlateralisation.

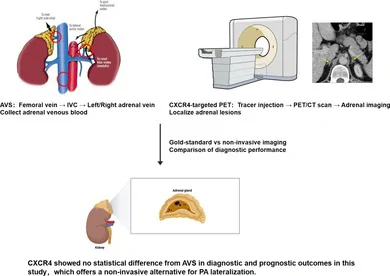

## 1 Introduction

Primary aldosteronism (PA) is the most common form of secondary hypertension and is characterised by unsuppressible aldosterone synthesis and secretion of the adrenal cortex [1]. Compared with essential hypertension, patients with PA have an increased risk of cardiovascular and renal morbidity, independent of blood pressure levels [2, 3] Early recognition and accurate subtype differentiation are therefore key points for PA management. Unilateral PA is usually surgically curable. Bilateral PA is not recommended for surgery, as it could lead to adrenal function loss and require life‐long hormone supplement therapy [4]. Adrenal Venous Sampling remains the reference standard for lateralisation of aldosterone hypersecretion [5, 6]. However, AVS is technically demanding and invasive. Failure rate ofcatheterisation, especially into the right adrenal vein, is reported to range between 3.5 [7] and 12% [8]. Successful sampling is highly dependent on operator expertise, and failure or inconclusive results are not uncommon, which limits its widespread application. These challenges have prompted the search for alternative or complementary diagnostic tools [9, 10].

Molecular imaging systems targeting the chemokine receptor CXCR4 ([^68^Ga]Pentixafor PET/CT) and CYP11B2 ([^11^C]Metomidate PET/CT, [^18^F]FMTO(fluorometomidate)) have recently emerged as novel noninvasive methods for adrenal lateralisation in PA. Preliminary studies suggest that CXCR4‐directed positron emission tomography (PET) may discriminate unilateral from bilateral disease, but evidence regarding its diagnostic accuracy and clinical utility remains limited [11]. Importantly, few studies with large sample sizes have directly compared CXCR4 imaging with AVS while incorporating postsurgical outcomes as endpoints.

In this retrospective study, we analysed a cohort of patients with confirmed PA who underwent lateralisation assessment either by AVS, CXCR4 imaging, or both. Postsurgical blood pressure control and biochemical cure were used as primary endpoints to evaluate the clinical impact of each diagnostic approach. In the subgroup with technically successful AVS and concurrent CXCR4 imaging, AVS was considered the reference standard to assess the diagnostic accuracy of CXCR4. This study aims to provide further evidence on the comparative value of AVS and CXCR4 imaging for lateralisation in PA [12, 13, 14, 15].

## 2 Methods

### 2.1 Data Resource and Study Population

This retrospective cohort study included patients with primary aldosteronism (PA) who underwent unilateral adrenalectomy (ADX) at West China Hospital, Sichuan University, between February 2023 and June 2025. Surgical indications are determined after lateralisation of aldosterone hypersecretion is confirmed by either AVS or CXCR4 PET/CT. If neither of the two examinations indicated unilateral dominant secretion, they were not included in this study. All participants provided written informed consent, and the study was approved by the Ethics Committee of West China Hospital, Sichuan University (Approval No. 2019, Review No. 692). Also, a retrospective analysis of 16 patients with primary aldosteronism (PA) who underwent both adrenal venous sampling (AVS) and CXCR4 PET/CT prior to unilateral adrenalectomy was also conducted.

### 2.2 Inclusion Criteria

(1) Diagnosis of PA was based on the screening and confirmatory protocols recommended by the European Society of Hypertension (ESH) and previous institutional studies ([16]). Patients with a screening aldosterone‐to‐renin ratio (ARR) > 2.4 (ng/dL)/(mIU/L) underwent either a captopril challenge test (CCT) or a seated saline infusion test (SSIT). PA was confirmed if plasma aldosterone concentration (PAC) exceeded 11 ng/dL in CCT or 10 ng/dL in SSIT [17]. (2) The presence of adrenal nodules ≥ 5 mm in diameter was verified by contrast‐enhanced adrenal computed tomography (CT). (3) Successful adrenal venous sampling (AVS) or CXCR4‐targeted molecular imaging was achieved. (4) Patients had a minimum of six months of postoperative follow‐up. Exclusion criteria: Patients were excluded if they (1) had severe hepatic or renal dysfunction; (2) had a history of malignanttumours; (3) experienced major cardiovascular events (e.g., myocardial infarction, stroke, or coronary surgery) within three months prior to enrollment; (4) were pregnant or breastfeeding; (5) required long‐term corticosteroid therapy; or (6) exhibited autonomous cortisol secretion not suppressed below 50 nmol/L after a 1 mg dexamethasone suppression test.

### 2.3 Classification and Diagnosis Method

The indication for unilateral ADX was determined following confirmation of the dominant aldosterone‐secreting side using either AVS or CXCR4‐directed molecular imaging. AVS was performed under digital subtraction angiography guidance at West China Hospital. Continuous intravenous adrenocorticotropic hormone (ACTH) infusion (25 IU bolus followed by sustained infusion) was administered to ensure stable hormonal secretion. Blood samples were collected from both adrenal veins and the inferior vena cava for cortisol and aldosterone measurement. Successful catheterisation was defined as a cortisol concentration ratio between the adrenal vein and the inferior vena cava > 3. The lateralisation index (LI), calculated as the aldosterone‐to‐cortisol ratio (A/C) between the two adrenal veins, was used to identify dominance. An LI > 4 was interpreted as unilateral aldosterone overproduction [18].

CXCR4‐targeted PET imaging was conducted in the Department of Nuclear Medicine, West China Hospital. A CXCR4‐targeted PET/CT examination was considered technically adequate when image quality permitted reliable visual and semiquantitative assessment of adrenal uptake in correlation with CT findings. Studies with severe motionartefacts, inadequate tracer distribution, or inability to localise adrenal uptake would be considered non‐diagnostic. No non‐diagnostic PET/CT examinations were observed in this cohort. After synthesis of ^68^Ga‐pentixafor, 3–5.5 mCi was intravenously administered, followed by adrenal PET/CT or PET/MRI scans at 40–60 min post‐injection. Within the area of ≥ 5 mm nodules confirmed by CT, unilateral aldosterone secretion was defined by any of the following criteria: Maximum standardised uptake value (SUV_max) of adrenal lesion > 6.5, lesion‐to‐liver SUV ratio (LLR) > 2.5, or lesion‐to‐contralateral adrenal SUV ratio (LAR) > 2.4 ([16]). Positive PET findings were defined as focal tracer uptake corresponding to a discrete adrenal nodule on CT rather than diffuse adrenal uptake. SUVmax was measured within the CT‐defined nodule whenever identifiable. If the uptake value increases but does not overlap with the clearly identified nodules on the CT scan, it is regarded as diffuse uptake and is not included in the unilateral positive judgement criteria.

PAC and direct renin concentration (DRC) were quantified using a LIAISON chemiluminescent immunoassay system (Diasorin, Italy). The intra‐assay and inter‐assay coefficients of variation for PAC were 2.1%–4.2% and 5.8%–10.8%, respectively, and for DRC were 2.1%–4.9% and 6.8%–13.0%. Reference ranges were as follows: PAC (recumbent 3–23.6 ng/dL, upright 3–35.3 ng/dL); DRC (recumbent 2.8–39.9 mIU/L, upright 4.4–46.1 mIU/L). Clinical data, including blood pressure, renal function (serum creatinine, eGFR), serum potassium, PAC, DRC, ARR, and antihypertensive drug use, were recorded six months postoperatively.

Formalin‐fixed paraffin‐embedded adrenal tissues were subjected to haematoxylin–eosin (HE) staining only. Immunohistochemical staining for CYP11B1 and CYP11B2 was not performed routinely and was carried out in just one representative case using an automated immunostainer. Primary antibodies against CYP11B1 (MABS502) and CYP11B2 (MABS1251) were obtained from Millipore. According to the HISTALDO consensus criteria, lesions were evaluated based on HE morphology, and a definitive classification of aldosterone‐producing adenoma (APA), micronodular hyperplasia, or diffuse hyperplasia was not performed due to the lack of routine IHC.

### 2.4 Follow‐Up Information and Postoperative Evaluation

Patients were routinely followed up for clinical and biochemical outcomes, including blood pressure, serum electrolytes, renal function, PAC, DRC, and ARR. Clinical success after surgery was evaluated based on the Primary Aldosteronism Surgical Outcome (PASO) criteria [19]. (1) Complete clinical success: Normotension (< 140/80 mmHg) without antihypertensive medication and maintenance of serum potassium > 3.5 mmol/L without supplementation. (2) Partial clinical success: Improved blood pressure control with reduced medication requirement and normokalemia. (3) Absent clinical success: No meaningful change in blood pressure or persistence of hypokalemia. Patients achieving complete or partial success were regarded as having an effective clinical response. Biochemical assessment: (1) Complete biochemical success: (a) postoperative orthostatic ARR ≤ 2.4 (ng/dL)/(mIU/L) with normal potassium; or (b) ARR > 2.4 but PAC < 11 ng/dL after CCT. (2) Partial biochemical success: ARR > 2.4 with PAC > 11 ng/dL after CCT, accompanied by > 50% reduction in PAC compared with baseline. (3) Absent biochemical success: Criteria for complete or partial success not met. Surgical cure was established when both clinical and biochemical complete success were achieved.

### 2.5 Statistical Analysis

Continuous variables with normal distributions were presented as mean ± standard deviation, whereas those with skewed distributions were expressed as median (interquartile range). Categorical variables were summarised as frequencies and percentages. Between‐group comparisons were conducted using the Kruskal–Wallis test for non‐normally distributed variables and the chi‐square test for categorical variables. Diagnostic performance metrics—including sensitivity, specificity, accuracy, positive predictive value (PPV), negative predictive value (NPV), and Cohen's κ for agreement—were calculated.

Diagnostic accuracy, sensitivity, specificity, and predictive values were calculated for both AVS and CXCR4 imaging. Agreement between the two diagnostic modalities was evaluated using Cohen's κ coefficient and McNemar's test. All analyses were two‐tailed, with *p* < 0.05 considered statistically significant. Statistical processing was performed using R software (version 4.2.2). This study followed the STROCSS reporting guideline.

The work has been reported in line with the STROCSS criteria [20].

## 3 Results

### 3.1 PA Patients Enrollment and Baseline Characteristics

**FIGURE 1:**
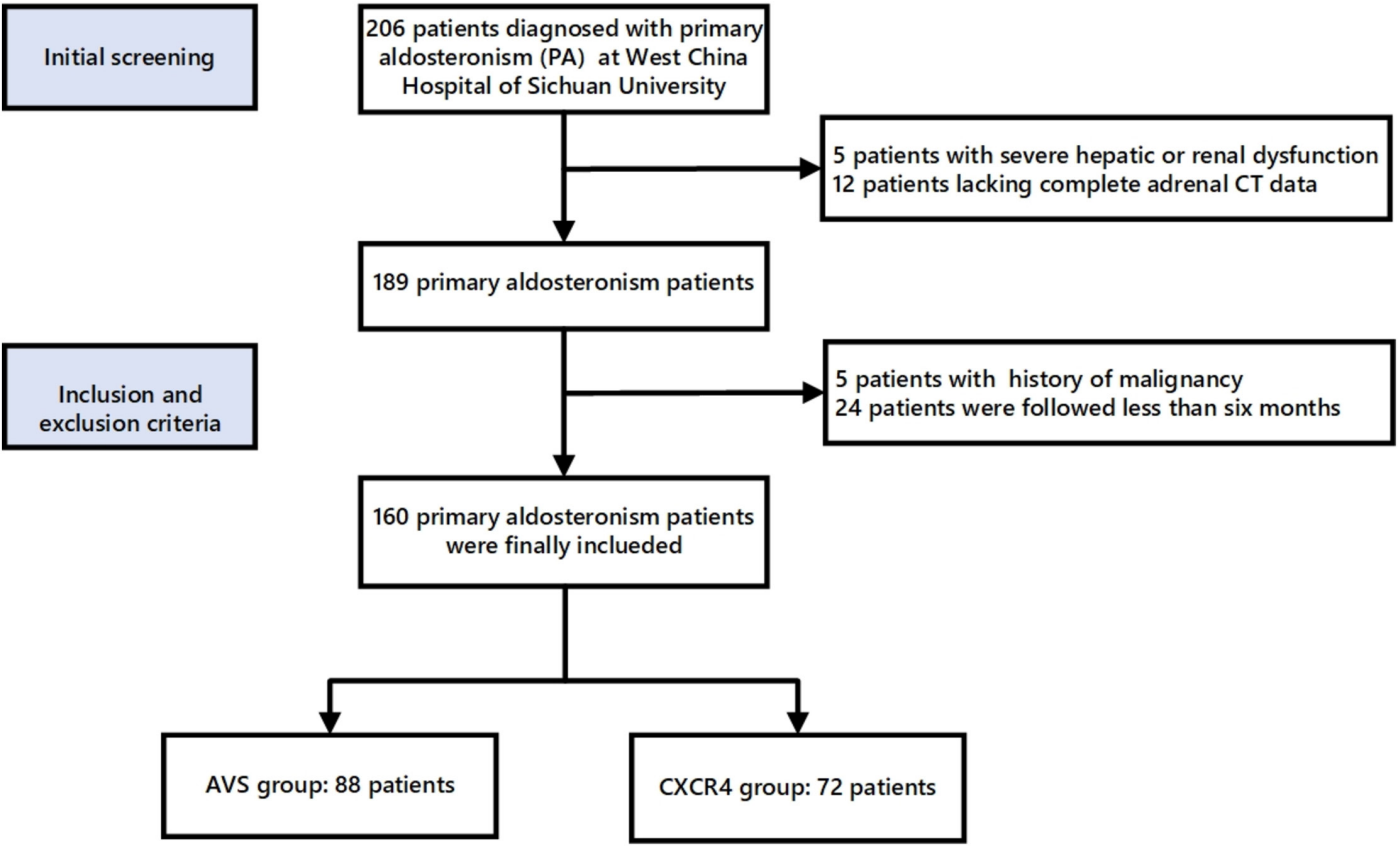
Flowchart of PA patients’ enrollment.

From February 2023 to June 2025, a total of 206 patients diagnosed with primary aldosteronism (PA) who provided written informed consent underwent unilateral adrenalectomy at the Department of Endocrinology and Metabolism, West China Hospital, Sichuan University. As illustrated in Figure 1, five patients with severe hepatic or renal dysfunction and twelve patients lacking complete adrenal CT data were excluded. In addition, five patients with a prior or suspected history of malignancy and 24 patients with a follow‐up period of less than six months were also excluded. Ultimately, 88 patients in the AVS group and 72 patients in the CXCR4 imaging group were included in the final analysis.

As shown in Table 1, baseline characteristics, including age, sex, BMI, blood pressure, serum creatinine, eGFR, PAC, DRC, ARR, and PAC after CCT/SSIT, showed no significant differences between the AVS and CXCR4 groups.

**TABLE 1:** Baseline characteristics for PA patients.

	Category/unit	Group		*p*
		AVS group	CXCR4 group	
		*n* = 88	*n* = 72	
Age (median [IQR])		49.00 [42.75, 56.00]	51.00 [39.75, 58.00]	0.966
Age class (%)	< 30	0 (0.00)	3 (4.17)	0.073
	< 40	12 (13.64)	15 (20.83)	
	< 50	35 (39.77)	17 (23.61)	
	< 60	28 (31.82)	23 (31.94)	
	≥ 60	13 (14.77)	14 (19.44)	
Metabolism diseases(%)	No	76 (86.36)	62 (86.11)	1
	Yes	12 (13.64)	10 (13.89)	
Surgery history (%)	No	77 (87.50)	60 (83.33)	0.602
	Yes	11 (12.50)	12 (16.67)	
Cardiovascular diseases (%)	No	82 (93.18)	67 (93.06)	1
	Yes	6 (6.82)	5 (6.94)	
Creatinine (median [IQR])		69.00 [59.50, 89.50]	77.50 [61.50, 95.00]	0.503
eGFR (median [IQR])		91.09 [82.26, 106.45]	96.32 [81.38, 105.17]	0.835
Hypertension years (median [IQR])		6.50 [2.00, 10.00]	5.50 [2.00, 10.00]	0.549
Maximum systolic blood pressure (mmHg) (median [IQR])		170.00 [160.00, 180.00]	170.00 [150.00, 180.00]	0.39
Maximum diastolic blood pressure (median [IQR])		110.00 [100.00, 111.25]	110.00 [100.00, 112.00]	0.995
Hypokalemia and related symptoms (%)	No	6 (6.90)	4 (5.97)	1
	Yes	81 (93.10)	63 (94.03)	
Kalemia level (median [IQR])		2.90 [2.54, 3.22]	2.71 [2.49, 3.15]	0.161
PAC before (median [IQR])		36.60 [21.90, 49.75]	37.30 [22.90, 57.20]	0.498
DRC before (median [IQR])		1.21 [0.61, 2.60]	1.49 [0.54, 3.80]	0.697
ARR before (median [IQR])		24.11 [11.57, 48.70]	19.63 [8.56, 66.96]	0.949
PAC after SSIT (median [IQR])		23.70 [14.30, 34.30]	28.45 [15.95, 46.10]	0.278
PAC after CCT (median [IQR])		26.30 [20.00, 36.70]	29.30 [22.35, 49.45]	0.178
Location (%)	Left	59 (67.05)	38 (52.78)	0.094
	Right	29 (32.95)	34 (47.22)	
Maximum diameter of adrenal nodules (cm) (%)	< 1	10 (12.20)	2 (2.82)	0.098
	< 2	60 (73.17)	57 (80.28)	
	≥ 2	12 (14.63)	12 (16.90)	

*Note:* Metabolism diseases, including: Hyperthyroidism, Diabetes, and Gout.

Abbreviations: ARR, aldosterone/renin ratio; CCT, captopril challenge test; DRC, direct renin concentration; eGFR, estimated glomerular filtration rate; PAC, plasma aldosterone concentration; SSIT, seated saline infusion test.

### 3.2 Remission of Patients in Both Groups After Unilateral Adrenalectomy

According to the standard definition of PASO, 93.1% (149/160) of all patients achieved biochemical success. The complete biochemical success rate was 84.1% (74/88) in the AVS group and 93.1% (67/72) in the CXCR4 group, and the difference was not statistically significant (*p* = 0.091). A total of 82.5% (132/160) of the patients achieved clinical success. The complete clinical success rate was 40.9% (36/88) in the AVS group and 30.3% (24/72) in the CXCR4 group, and the difference was not statistically significant (*p* = 0.412).

Analysis of variance (ANOVA) was performed to compare the biochemical and clinical outcomes between the two groups at 1 month and 6 months after surgery (Table 2). In terms of biochemical outcomes, there were no significant differences in DRC, PAC, ARR or serum potassium between the two groups after surgery.

**TABLE 2:** Biochemical outcomes between the two groups at 1 month and 6 months follow‐up after surgery.

*n*	Unit	AVS group	CXCR4 group	*p*
		88	72	
Surgical complication (%)		85 (96.59)	70 (97.22)	1
		3 (3.41)	2 (2.78)	
K (1 month) (median [IQR])	(mmol/L)	4.23 [3.84, 4.46]	4.32 [3.81, 4.53]	0.494
PAC (1 month) (median [IQR])	(ng/dl)	8.11 [5.36, 12.43]	7.56 [5.48, 11.75]	0.841
DRC (1 month) (median [IQR])	(mIU/L)	7.78 [3.04, 19.13]	9.29 [3.35, 23.65]	0.695
ARR (1 month) (median [IQR])	(ng/dl)/(mIU/L)	1.04 [0.48, 2.91]	0.97 [0.32, 2.32]	0.289
K (6 months) (median [IQR])	(mmol/L)	4.43 [4.17, 4.74]	4.32 [4.09, 4.54]	0.025
PAC (6 months) (median [IQR])	(ng/dl)	10.80 [4.93, 14.00]	7.64 [4.71, 13.60]	0.21
DRC (6 months) (median [IQR])	(mIU/L)	12.09 [4.78, 20.09]	12.46 [5.35, 30.50]	0.557
ARR (6 months) (median [IQR])	(ng/dl)/(mIU/L)	0.70 [0.48, 1.08]	0.70 [0.28, 1.46]	0.246

### 3.3 Diagnostic Performance of CXCR4 Compared With AVS

The study cohort also included 16 PA patients who underwent both AVS and CXCR4 PET/CT. Using AVS as the gold standard, CXCR4 PET/CT demonstrated a sensitivity of 70.0% (7/10), specificity of 83.3% (5/6), and an overall diagnostic accuracy of 75.0% (12/16) for detecting unilateral aldosterone hypersecretion. The positive predictive value (PPV) was 87.5%, and the negative predictive value (NPV) was 62.5%. Agreement between CXCR4 PET/CT and AVS was moderate (Cohen's κ = 0.54), and McNemar's test indicated no significant difference. Furthermore, utilising postoperative clinical and biochemical remission as the reference standard, the lateralisation accuracy of the two examinations was validated: Twelve cases had consistent AVS positioning results with the cured side after surgery, with an accuracy rate of 75%. Ten cases were judged correctly by CXCR4 PET/CT, with an accuracy rate of 62.5%. There was no statistically significant difference in the accuracy rates of the two methods (*p* > 0.05). This indicates that there is no significant difference in the positioning accuracy of the two methods, and both can effectively guide surgical decisions.

### 3.4 Representative Case of Multi‐Nodular Primary Aldosteronism Evaluated by CXCR4 PET/CT and AVS

**FIGURE 2:**
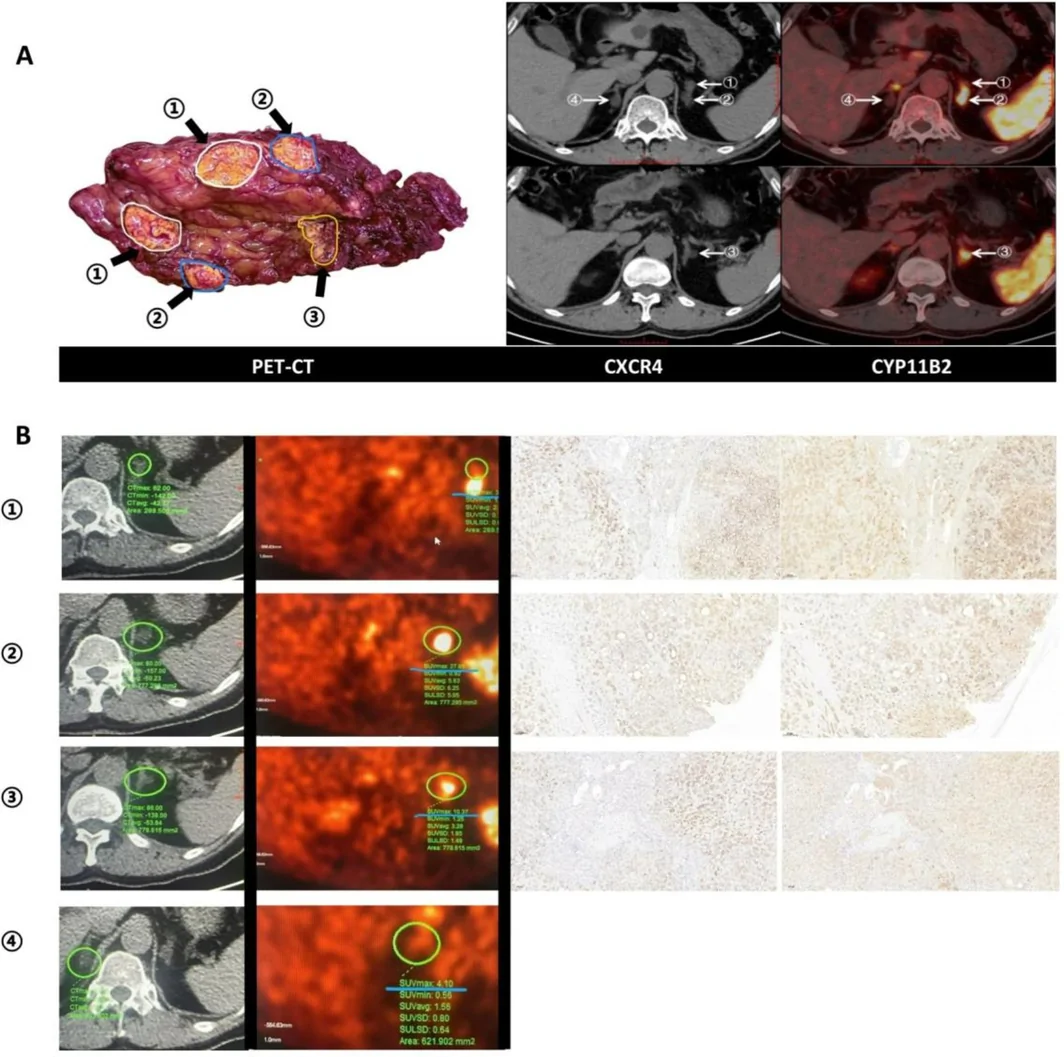
Concordant findings of CXCR4 PET/CT and histopathology in a patient with multi‐nodular primary aldosteronism.

Biochemical screening of a 62‐year‐old male with long‐standing hypertension and incidentally discovered bilateral adrenal nodules revealed suppressed renin and elevated aldosterone (PAC 15.7 ng/dL, DRC 0.61 uIU/mL; ARR 25.7), and positive saline infusion and captopril suppression tests confirmed autonomous aldosterone secretion. Cross‐sectional imaging identified three left adrenal nodules (①: 1.0*0.8 cm; ②: 1.6*1.4 cm; ③: 1.4*1.2 cm) and one right adrenal nodule (④: 1.8*1.1 cm). Under continuous ACTH infusion, AVS showed a cortisol‐corrected aldosterone lateralisation index of 8.5, indicating left‐sided aldosterone hypersecretion. Then, CXCR4‐targeted ^68^Ga‐pentixafor PET/CT demonstrated heterogeneous tracer uptake: Left nodules ② and ③ showed markedly elevated SUVmax (② 27.85; ③ 10.37), lesion‐to‐liver ratio (LLR ②14.81; ③ 5.52) and lesion‐to‐adrenal ratio (LAR ② 6.89 vs. ③ 2.57), consistent with functional aldosterone‐producing adenomas, whereas nodule ① (SUVmax 4.0) and the right nodule ④ (SUVmax 5.43; LLR 2.89; LAR 1.6) had low uptake, consistent with nonfunctional lesions (Figure 2A). The patient underwent left adrenalectomy. Histopathology confirmed multiple adrenocortical adenomas; immunohistochemistry showed strong CYP11B2 and CXCR4 positivity in all resected nodules (①–③), confirming them as aldosterone‐producing adenomas (Figure 2B). Postoperatively, the patient achieved biochemical cure: At 4 weeks, serum potassium normalised (4.2 mmol/L), and renin and aldosterone were suppressed off antihypertensives (DRC 2.9 uIU/mL, PAC 8.1 ng/dL); at 1 and 6 months follow‐up, he remained normokalemic and normotensive without medications, corresponding to complete biochemical remission and clinical success by PASO criteria.

Due to the retrospective design of this study and the limitation of sample size, quantitative statistical analysis was not conducted for the multi‐nodule subgroup. However, typical cases and postoperative pathological results can directly indicate the limitations of molecular imaging in this type of lesion.

## 4 Discussion

Our data indicate that CXCR4‐targeted ^68^Ga‐pentixafor PET/CT and adrenal venous sampling (AVS) show no statistical difference for subtype classification of primary aldosteronism and for predicting biochemical and clinical remission after unilateral adrenalectomy. However, this result should be interpreted with caution: This study was not designed as an equivalence or non‐inferiority trial. The sample sizes of the two groups were limited, and the absence of statistically significant differences might be due to insufficient statistical power. Therefore, it cannot be directly concluded that the two methods are equivalent.

CXCR4 PET/CT also demonstrated moderate agreement with AVS in lateralising aldosterone‐producing lesions, with high PPV but a slightly reduced sensitivity. The false‐negative cases likely reflect small, multi‐nodular, or heterogeneous lesions that are challenging to detect with PET imaging. These findings suggest that while CXCR4 PET/CT is a promising non‐invasive alternative forlocalisation, careful interpretation is warranted in cases with borderline uptake or complex adrenal morphology. Complementary evaluation with AVS may still be necessary in select patients to avoid under‐treatment.

Furthermore, a representative case in our series highlighted an important limitation: Imaging and histopathology were not fully concordant in a multinodular adrenal gland. Although only two nodules exhibited markedly increased tracer uptake on PET/CT, all resected lesions proved CYP11B2‐positive on immunohistochemistry. This discordance suggests that CXCR4 uptake does not always map exactly to aldosterone synthase activity at the lesion level. A comprehensive judgement should be made in combination with AVS or pathological features, which is conducive to improving the accuracy of diagnosis. Factors that may explain false‐negative imaging findings include intralesional heterogeneity of receptor expression, variable receptor density among neighbouring nodules, partial‐volume effects for small lesions, and technical limits of semiquantitative metrics. Furthermore, the expression of CXCR4 receptors in adrenal cortical lesions may be regulated by the local hormonal microenvironment and genetic background of the lesion itself. This could lead to the separation between receptor expression level and aldosterone synthesis function, and the potential regulatory mechanism needs to be further verified by basic experimental research. And this study only elaborates on this issue through a typical case. In the future, a larger sample study is needed to specifically explore the consistency of the two methods in cases of multiple nodules.

Clinically, these observations argue for caution when interpreting CXCR4 PET/CT in patients with multiple or small adrenal nodules. In straightforward, solitary lesions or when AVS is infeasible, CXCR4 imaging can reliably guide management and provide accurate lateralisation evidence for surgical decision‐making. However, in complex multinodular cases or when PET/CT results are equivocal, AVS—or a combined diagnostic strategy—remains important to confirm lateralisation before surgery. This comprehensive strategy can integrate the non‐invasiveness and high positive predictive value of CXCR4 PET/CT with the high accuracy of AVS, which is expected to further improve the diagnostic efficiency of PA lateralisation and reduce the risks of misdiagnosis and inappropriate treatment.

Compared with the previous preliminary studies on CXCR4 molecular imaging for PA, this research has significant clinical value. We use postoperative biochemical cure and clinical blood pressure control as the primary endpoints, and evaluate the diagnostic value of these two techniques from the perspective of prognostic guidance, which is more in line with the clinical demand of PA diagnosis for guiding treatment and judging outcomes. Some previous studies have reported different sensitivities and specificities of CXCR4 PET/CT, which may be related to the differences in research population, imaging quantitative threshold settings and detection equipment. Currently, the clinical application of CXCR4 PET/CT still lacks unified standardised diagnostic criteria, and the relevant indicators need to be further confirmed through multi‐centre large‐sample prospective studies.

This study has several limitations. First, its retrospective design and modest sample size introduce potential selection bias: Choice of diagnostic modality was influenced by patient preference, logistics, and perceived procedural risk, and younger patients appeared more likely to opt for CXCR4 imaging, which may affect the generalisability of the research results. Second, follow‐up assessments did not systematically include postoperative cortisol dynamics or renal function parameters, which could affect outcome interpretation. Third, routine pathological examination was limited to HE staining without subtype classification by CYP11B2 immunohistochemistry. As a result, potential imbalance in pathological characteristics between groups could not be assessed, which represents an important limitation of this retrospective study. In addition, this study did not conduct in‐depth correlation analyses of the expression level of the CXCR4 receptor with clinical pathological indicators such as tumour size and pathological type, and the molecular mechanism of CXCR4 in the occurrence and development of aldosterone‐secreting lesions still needs further clarification through a combination of basic research and clinical research. Future work should prospectively validate standardised PET quantification thresholds (e.g., SUVmax, LLR, LAR), incorporate molecular and histopathological correlates, and evaluate hybrid algorithms (molecular imaging plus selective sampling or radiomics) in larger, ideally randomised cohorts. All the patients in this study had clearly visible nodules in their adrenal CT scans (with the maximum diameter of ≥ 5 mm), and those with negative CT results for PA or microadenomas (< 5 mm) were not included. Therefore, the conclusion of this study is applicable only to PA patients with identifiable nodules on CT. The diagnostic value for CT‐negative PA and microadenomas still requires further research.

## 5 Conclusion

CXCR4‐directed ^68^Ga‐pentixafor PET/CT showed no statistical difference in diagnostic and prognostic performance compared to adrenal venous sampling in patients with primary aldosteronism. As a safe and noninvasive technique, it may serve as an alternative for cases where AVS is impractical. However, the observed discordance between CXCR4 uptake and histopathology in multinodular disease highlights that molecular imaging alone cannot fully capture aldosterone secretion heterogeneity. This conclusion still requires more cases for verification.

## Author Contributions

**Shengzhuo Liu:** writing – original draft, conceptualization, supervision. **Yifan Zhang:** writing – review and editing, project administration. **Huijun Zhou:** writing – review and editing, validation. **Xiaoyang Liu:** formal analysis, data curation. **Yuchun Zhu:** conceptualization, funding acquisition. **Sikui Shen:** visualization, validation.

## Funding

This work was supported by the Science and Technology Department of Sichuan Province (2024nsfsc1612) and the Postdoctoral Fund of West China Hospital (2023hxbx115). This work was supported by the National Natural Science Foundation of China (Grant No. 82573700).

## Disclosure

The authors have nothing to report.

## Consent

The authors have nothing to report.

## Conflicts of Interest

The authors declare no conflicts of interest.

## Data Availability

The data that support the findings of this study are available on request from the corresponding author. The data are not publicly available due to privacy or ethical restrictions.

## References

[1] J. M. Brown , M. Siddiqui , D. A. Calhoun , et al., “The Unrecognized Prevalence of Primary Aldosteronism,” Annals of Internal Medicine 173 (2020): 10–20, 10.7326/M20-0065. 32449886 PMC7459427

[2] K. W. Choy , P. J. Fuller , G. Russell , Q. Li , M. Leenaerts , and J. Yang , “Primary aldosteronism,” BMJ 377 (2022): e065250, 10.1136/bmj-2021-065250. 35443988

[3] Primary aldosteronism – a multidimensional syndrome , “Nature Reviews Endocrinology [WWW Document] 2022,” accessed August 10, 2025, https://www.nature.com/articles/s41574‐022‐00730‐2. 10.1038/s41574-022-00730-2 36045149

[4] M. Naruse , T. Katabami , H. Shibata , et al., “Japan Endocrine Society Clinical Practice Guideline for the Diagnosis and Management of Primary Aldosteronism 2021,” Endocrine Journal 69 (2022): 327–359, 10.1507/endocrj.EJ21-0508. 35418526

[5] M. Reincke , I. Bancos , P. Mulatero , U. I. Scholl , M. Stowasser , and T. A. Williams , “Diagnosis and Treatment of Primary Aldosteronism,” Lancet Diabetes and Endocrinology 9 (2021): 876–892, 10.1016/S2213-8587(21)00210-2. 34798068

[6] S. Yang , Z. Du , X. Zhang , et al., “Corticotropin Stimulation in Adrenal Venous Sampling for Patients With Primary Aldosteronism,” JAMA Network Open 6 (2023): e2338209, 10.1001/jamanetworkopen.2023.38209. 37870836 PMC10594148

[7] S. O. Trerotola , M. Asmar , Y. Yan , D. L. Fraker , and D. L. Cohen , “Failure Mode Analysis in Adrenal Vein Sampling: A Single‐Center Experience,” Journal of Vascular and Interventional Radiology 25 (2014): 1611–1619, 10.1016/j.jvir.2014.06.029. 25130306

[8] T. Wannachalee , E. Caoili , K. Nanba , et al., “The Concordance Between Imaging and Adrenal Vein Sampling Varies With Aldosterone‐Driver Somatic Mutation,” Journal of Clinical Endocrinology and Metabolism 105 (2020): e3628–e3637, 10.1210/clinem/dgaa482. 32717082 PMC7437239

[9] V. A. Ohannesian , D. Rajchenberg , I. R. Menezes , et al., “Pooled Diagnostic Accuracy of 68Ga‐Pentixafor PET/CT for Aldosterone‐Producing Lesions in Primary Aldosteronism,” Clinical Imaging, 131 (2026): 110743, 10.1016/j.clinimag.2026.110743. Epub ahead of print. PMID: 41653499. 41653499

[10] Y. Peng , F. Chen , R. Yao , et al., “The Value of Targeted CXCR4 18F‐AlF‐NOTA‐Pentixafor PET/CT for Subtyping Primary Aldosteronism,” Front Endocrinol (Lausanne) 16 (2025): 1533295, 10.3389/fendo.2025.1533295. 40084145 PMC11903271

[11] Y. Zheng , T. Long , N. Peng , et al., “The Value of Targeting CXCR4 With 68Ga‐Pentixafor PET/CT for Subtyping Primary Aldosteronism,” Journal of Clinical Endocrinology and Metabolism 109, no. 1 (2023): 171–182, 10.1210/clinem/dgad421. 37477496

[12] S. J. Gandhi , N. P. Kunder , S. Gupta , and R. Kute , “Gallium‐68‐Pentixafor PET/CT for Subtyping Diagnosis of Primary Aldosteronism: A Pictorial Essay,” Indian Journal Of Nuclear Medicine 40, no. 3 (2025): 161–165, 10.4103/ijnm.ijnm_151_24. 40927148 PMC12416569

[13] Z. Shu , Y. He , T. Long , et al., “Is CXCR4‐Targeted 68Ga‐Pentixafor PET/CT a Reliable AVS‐Free Modality for Surgical Decision‐Making and Prognostic Prediction in Primary Aldosteronism With Bilateral Adrenal Lesions?,” EJNMMI Research 15, no. 1 (2025): 111, 10.1186/s13550-025-01309-4. PMID: 40875090; PMCID: PMC12394099. 40875090 PMC12394099

[14] X. Wei , F. Wu , H. Dong , et al., “68Ga‐Pentixafor PET/CT in the Localization Diagnosis of Primary Aldosteronism Concurrent Subclinical Cushing's Syndrsome: Two Case Reports,” Endocrine 85, no. 3 (2024): 1398–1406, 10.1007/s12020-024-03865-6. Epub 2024 Jun 24. PMID: 38914747. 38914747

[15] G. Zheng , S. Liu , Y. Gao , et al., “68 ga‐Pentixafor PET/CT Versus Adrenal Venous Sampling in Guiding Surgical Management for Primary Aldosteronism: A Prospective Cohort Study on Postoperative Outcomes,” International Journal of Surgery 112, no. 1 (2026): 844–853, 10.1097/JS9.0000000000003483. Epub 2025 Sep 9. PMID: 40932337; PMCID: PMC12825820. 40932337 PMC12825820

[16] L. Tan , T. Chen , W. Zhang , et al., “CXCR4‐Directed PET With 68Ga‐Pentixafor Versus Adrenal Vein Sampling for the Diagnosis of Unilateral Primary Aldosteronism,” Endocrine 89 (2025): 603–613, 10.1007/s12020-025-04236-5. 40388084 PMC12289708

[17] J. W. Funder , R. M. Carey , F. Mantero , et al., “The Management of Primary Aldosteronism: Case Detection, Diagnosis, and Treatment: An Endocrine Society Clinical Practice Guideline,” Journal of Clinical Endocrinology and Metabolism 101 (2016): 1889–1916, 10.1210/jc.2015-4061. 26934393

[18] N. Younes , S. Larose , I. Bourdeau , E. Therasse , and A. Lacroix , “Role of Adrenal Vein Sampling in Guiding Surgical Decision in Primary Aldosteronism,” Experimental and Clinical Endocrinology & Diabetes 131 (2023): 418–434, 10.1055/a-2106-4663. 37567230

[19] T. A. Williams , J. W. M. Lenders , P. Mulatero , et al., “Outcomes After Adrenalectomy for Unilateral Primary Aldosteronism: An International Consensus on Outcome Measures and Analysis of Remission Rates in an International Cohort,” Lancet Diabetes & Endocrinology 5 (2017): 689–699, 10.1016/S2213-8587(17)30135-3. 28576687 PMC5572673

[20] G. Mathew , R. Agha , J. Albrecht , et al., “STROCSS 2021: Strengthening the Reporting of Cohort, Cross‐Sectional and Case‐Control Studies in Surgery,” Annals of Medicine and Surgery 72 (2021): 103026, 10.1016/j.amsu.2021.103026. 34820121 PMC8599107

